# Variable-State-Dimension Kalman-Based Filter for Orientation Determination Using Inertial and Magnetic Sensors

**DOI:** 10.3390/s120708491

**Published:** 2012-06-25

**Authors:** Angelo Maria Sabatini

**Affiliations:** The BioRobotics Institute, Scuola Superiore Sant'Anna, Piazza Martiri della Libertà 33, Pisa 56127, Italy; E-Mail: sabatini@sssup.it; Tel.: +39-05-088-3415; Fax: +39-05-088-3101

**Keywords:** inertial/magnetic sensing, sensor fusion, orientation determination, Kalman filters

## Abstract

In this paper a quaternion-based Variable-State-Dimension Extended Kalman Filter (VSD-EKF) is developed for estimating the three-dimensional orientation of a rigid body using the measurements from an Inertial Measurement Unit (IMU) integrated with a triaxial magnetic sensor. Gyro bias and magnetic disturbances are modeled and compensated by including them in the filter state vector. The VSD-EKF switches between a quiescent EKF, where the magnetic disturbance is modeled as a first-order Gauss-Markov stochastic process (GM-1), and a higher-order EKF where extra state components are introduced to model the time-rate of change of the magnetic field as a GM-1 stochastic process, namely the magnetic disturbance is modeled as a second-order Gauss-Markov stochastic process (GM-2). Experimental validation tests show the effectiveness of the VSD-EKF, as compared to either the quiescent EKF or the higher-order EKF when they run separately.

## 1. Introduction

Accurate determination of the three-dimensional (3D) orientation of rigid bodies is important in several technical fields, including robotics and virtual reality [1], human motion analysis [2] and pedestrian navigation [3]. One popular approach to the design of orientation trackers in these applications is based on the physical principles of inertial navigation [4,5].

Usually, a fully integrated inertial measurement unit (IMU) consists of three vector-sensors, namely an accelerometer, a gyro, and a magnetic sensor; each of them has three mutually orthogonal sensitive axes. The 3D orientation can be computed by time-integrating the gyro output from initial conditions that are given by the aiding sensors (accelerometer and magnetic sensor). The gyro integration errors tend to grow unbounded over time because of time-varying biases and wideband measurement noise superimposed to gyro output. On the other hand, the accelerometer can provide drift-free inclination estimates by sensing the gravity field; the magnetic sensor, which would sense the Earth's magnetic field, helps providing drift-free heading estimates. However, the interpretation of the accelerometer output is critical because of the presence of the acceleration components related to the body motion [6]; moreover, additional artificial magnetic fields determining magnetic anomalies or distortions lead to severe errors in the heading estimates [7–9].

Sensor fusion techniques are needed in order for the aiding sensors to help mitigate the gyro integration errors, while the errors incurred by the aiding sensors are mitigated using the gyro output [10]. Extended Kalman filters (EKF) are by far the most common tools used to achieve this goal [2,11,12]. In our previous research, a quaternion-based EKF was developed; the angular velocity was taken as a control input and compensation of gyro bias and magnetic disturbance was implemented using state-augmentation [12,13].

State-augmentation is a useful technique to estimate the state vector of interest, *i.e.*, the orientation representation, jointly with a number of variables that are uncertain, e.g., the sensor errors [14,15]. The state vector is augmented with a vector containing the uncertain variables, and the model of the uncertain variables are accounted for in the state transition model before applying the Kalman filter equations. For instance, a popular approach to magnetic disturbance compensation assumes that the deviations from the local reference magnetic field, henceforth also called magnetic distortions, are first-order Gauss-Markov (GM-1) stochastic vector processes [2,13,16].

Because of some difficulties in tracking abrupt magnetic distortions [16], the GM-1 model is better suited in our view to describe the time-rate of change of the magnetic field, namely the magnetic distortions are modeled with a second-order Gauss-Markov model (GM-2) model. We are unaware of any previous work modeling magnetic distortions in this manner. In this paper, we present a variable-state-dimension (VSD) approach to the development of a Kalman-filter-based orientation tracker. The VSD-EKF switches between the quiescent EKF and the higher-order EKF, where the model of the magnetic distortion is, respectively, GM-1 and GM-2; the switching rule is when the fading memory average of the normalized filter innovation exceeds a given threshold. The switching back to the quiescent EKF occurs when the time-rate of change of the magnetic field is detected small. Under quiet magnetic conditions using the quiescent EKF may yield maximum estimation accuracy: no information is wasted on estimating components that are zero or small. Experimental validation tests show the effectiveness of the proposed VSD-EKF in magnetically perturbed environments, as compared to either the quiescent EKF or the higher-order EKF when they run separately.

## 2. Method

The VSD-EKF estimates the following state variables: the quaternion between the body-fixed frame, *i.e.*, the B-frame, whose axes are assumed aligned with the triad of IMU sensitivity axes, and the Earth-fixed frame, *i.e.*, the E-frame; the gyro-bias and the components of the magnetic distortion, expressed in the E-frame, including their time-rates of change when the higher-order EKF is invoked.

### 2.1. Kinematic Equations

The time-derivative 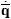 of the quaternion **q̄**=[**q***^T^* q_4_]*^T^* and the angular velocity **ω** = [*p q r*]*^T^* of the B-frame relative to the E-frame, resolved in the B-frame, are related by the following system of differential equations[17,18], where the time argument is dropped for notational convenience:

(1) $$\dot{\bar{\text{q}}}=\frac{1}{2}\left[\begin{array}{cc} -\left[\omega \times \right] & \omega \\ -\omega^{T} & 0 \end{array}\right]\bar{\text{q}}=\Omega \left(\omega \right)\bar{\text{q}}.$$

In (1) the operator:

(2) $$\left[\omega \times \right]=\left[\begin{array}{ccc} 0 & -r & q\\ r & 0 & -p\\ -q & p & 0 \end{array}\right]$$

is the standard vector cross product.

The discrete-time equivalent of (1) is given by:

(3) $$\bar{\text{q}}\left(t_{k}\right)=\Phi_{k-1}\bar{\text{q}}\left(t_{k-1}\right),$$

with:

(4) $$\Phi_{k-1}={\text{I}}_{4}\cdot \cos\left(\left\|{\text{u}}_{k-1}\right\|/2\right)+\Omega \left(\omega \left(t_{k-1}\right)\right)\cdot \frac{\sin\left(\left\|{\text{u}}_{k-1}\right\|/2\right)}{\left\|{\text{u}}_{k-1}\right\|/2},$$

where **I***_n_* is the *n* × *n* identity matrix (*n* = 4) and ‖·‖denotes the standard vector norm. If the angular velocity is assumed constant during the sampling interval *T_s_* = *t_k_* − *t_k_*_−1_, we have:

(5) $${\text{u}}_{k-1}=\int_{t_{k-1}}^{t_{k}}\omega \left(\tau \right)d\tau \approx \omega \left(t_{k-1}\right)T_{s}.$$

In this paper the IMU signals are sampled at *T_s_* = 0.01 s.

### 2.2. Modeling the Magnetic Distortion

The conditions for accurate orientation determination using a magnetic sensor are that the magnetic field be homogeneous and accurately known. The homogeneity is difficult to achieve [7–9]. The magnetic field can thus unpredictably change in direction and magnitude when the IMU moves relative to the environment. We propose two stochastic models for describing the magnetic distortion *^h^***b**, *i.e.*, the difference between the magnetic field and its vector value in a given point at a given time—the local reference magnetic field **h**—both expressed in the E-frame.

The GM-1 model assumes that *^h^***b** is the realization of an exponentially time-correlated vector stochastic process whose components are statistically independent:

(6) b˙h=-αLbh+wL,

where *α_L_* is the reciprocal of the correlation time constant *τ_L_* and **w***_L_* is white Gaussian noise, with zero mean and covariance matrix ∑Lh=I3⋅σb2L.

The GM-2 model assumes that the time-rate of change of *^h^***b** is the realization of a GM-1 vector stochastic process with statistically independent components:

(7) b¨h=-αHb˙h+wH,

where *α_H_* is the reciprocal of the correlation time constant *τ_H_*, and **w***_H_* is white Gaussian noise, with zero mean and covariance matrix ∑Hh=I3⋅σb2H. Henceforth, we refer to σb2H as the strength of the GM-2 model driving noise.

### 2.3. IMU Sensor Modeling

In response to body angular velocity **ω***_b_*, acceleration (gravity field **g** and body acceleration **a***_b_*), and magnetic field *^s^***h** (local magnetic field **h** and magnetic distortion *^h^***b**), the measured outputs of the IMU sensors can be written as [10]:

(8) {ωm=Ggωb+bg+vgam=GaCEB(-g+ab)+ba+vamm=GmCEB(h+bh)+bm+vm.

The orientation matrix CEB moves vector representations from the E-frame to the B-frame; *^g^***G**, *^a^***G**, *^m^***G** are the matrices of the scale factors (ideally equal to the identity matrix **I**_3_); *^g^***b**, *^a^***b**, *^m^***b** are the bias vectors (ideally, they are zero); *^g^***v**, *^a^***v**, *^m^***v** are uncorrelated white Gaussian measurement noises, with zero mean and covariance matrix $\sum_{g}={\text{I}}_{3}\cdot \sigma_{g}^{2},\sum_{a}={\text{I}}_{3}\cdot \sigma_{a}^{2}$ and $\sum_{m}={\text{I}}_{3}\cdot \sigma_{m}^{2}$. Equation (8) is a model that does not account for additional error sources, such as cross-axis sensitivity, gyro *g*-sensitivity, nonlinearity, hysteresis and misalignment [19]. Finally, we also assume for simplicity that the tracked objects are slowly moving, namely **a***_b_* ≈ **0**.

The deterministic errors associated with inertial and magnetic sensors can be compensated using the procedures described in [19,20], respectively, leaving the time varying biases, in particular of gyros, as the residual errors. In line with a common practice [2,10,14,15], we model the gyro-bias vector as the realization of a random-walk vector stochastic process with statistically independent components:

(9) b˙g=wg.

where **w***_g_* is white Gaussian noise, with zero mean and covariance matrix ∑g=I3⋅hσb2d.

### 2.4. Filter Development

The VSD-EKF adopts two state models: the quiescent state model, where the state vector is **x**=[**q̄***^T h^***b***^T g^***b***^T^*]*^T^* and the magnetic distortion is GM-1, and the higher-order state model, where the state vector is **x**=[**q̄***^T h^***b***^T h^***ḃ***^T g^***b***^T^*]*^T^* and the magnetic distortion is GM-2. The quiescent EKF is fully developed in [13]. The continuous-time system equations of the higher-order EKF are written as follows:

(10) {q¯˙=Ω(ω)q¯b¨h=-αHb˙h+wHb˙g=wg.

The discrete-time model allows employing the sampled measurements of the IMU for propagating the state vector:

(11) [q¯kbkhb˙khbkg]=Fk-1[q¯k-1bk-1hb˙k-1hbk-1g]+[wk-1q03×1wk-1hwk-1g]

where:

(12) $${\text{F}}_{k-1}=\left[\begin{array}{cccc} \Phi_{k-1} & 0_{4\times 3} & 0_{4\times 3} & 0_{4\times 3}\\ 0_{3\times 4} & {\text{I}}_{3} & {\text{I}}_{3}\cdot \frac{1-\exp\left(-\alpha_{H}T_{s}\right)}{\alpha_{H}} & 0_{3\times 3}\\ 0_{3\times 4} & 0_{3\times 3} & {\text{I}}_{3}\cdot \exp\left(-\alpha_{H}T_{s}\right) & 0_{3\times 3}\\ 0_{3\times 4} & 0_{3\times 3} & 0_{3\times 3} & {\text{I}}_{3} \end{array}\right]$$

and:

(13) $$\Phi_{k-1}={\text{I}}_{4}\cdot \cos\left(\left\|\tilde{\omega }\left(t_{k-1}\right)\right\|T_{s}/2\right)+\Omega \left(\tilde{\omega }\left(t_{k-1}\right)\right)\cdot \frac{\sin\left(\left\|\tilde{\omega }\left(t_{k-1}\right)\right\|T_{s}/2\right)}{\left\|\tilde{\omega }\left(t_{k-1}\right)\right\|T_{s}/2}.$$

In (13) the angular velocity **ω̃** = **ω***_m_* − *^g^***b** is taken as the control input.

The process noise component *^q^***w***_k_*_−1_ describes how the gyro measurement noise enters the state model through a quaternion-dependent linear transformation:

(14) wqk-1=-Ts2Ξ(q¯k-1)vgk-1

and the matrix **Ξ**(**q̄**) is given by:

(15) $$\Xi \left(\bar{\text{q}}\right)=\left[\begin{array}{c} {\text{I}}_{3}\cdot q_{4}+\left[\text{q}\times \right]\\ -{\text{q}}^{T} \end{array}\right].$$

Since the process noise components *^q^***w***_k_*_−1_, *^h^***w***_k_*_−1_, *^g^***w***_k_*_−1_ are assumed to be uncorrelated, the process noise covariance matrix **Q***_k_*_−1_ has the following structure [21,22]:

(16) Qk-1=[(I4⋅trace(M)-M)⋅σg2(Ts2)203×303×306×3QH03×303×303×3I3⋅σbg2Ts]QH=[Q11HQ12HQH12TQ22H]Q11H=I3⋅4exp(-αHTs)-3-exp(-2αHTs)+2αHTs2αH3σH2Q12H=I3⋅exp(-2αHTs)+1-2exp(2αHTs)2αH2σH2Q22H=I3⋅1-exp(-2αHTs)2αHσH2

where:

(17) $$\text{M}={\bar{\text{q}}}_{k-1}{\bar{\text{q}}}_{k-1}^{T}+{\text{P}}_{k-1}^{q}.$$

**q̄***_k_*_−1_ and ${\text{P}}_{k-1}^{q}$ denote, respectively, the expectation and the covariance matrix of the quaternion component of the state vector [22].

The measurement equations can be written as follows:

(18) [am,kmm,k]=[CEB(q¯k)03×303×3CEB(q¯k)][-gbsk]+[vakvmk].

The rotation matrix can be derived from the quaternion [10]:

(19) CEB(q¯)=I3-2q4[q×]+2[q×]2.

The measurement equations are non-linear, hence we need to compute their Jacobian matrix **H**, as prescribed by the EKF linearization process:

(20) Hk=[ψ(q¯k,-g)03×303×303×3ψ(q¯k,bs)CEB(q¯k)03×303×3],

where, for a generic vector **p**, we define:

(21) ψ(q¯,p)=∂∂q¯CEB(q¯)p.

The detailed calculation of (21) is illustrated in [13]. The measurement noise covariance matrix can be expressed directly in terms of the statistics of the measurement noise affecting each sensor:

(22) $$\text{R}=\left[\begin{array}{cc} \sum_{a} & 0_{3\times 3}\\ 0_{3\times 3} & \sum_{m} \end{array}\right].$$

Vector selection methods for measurement noise covariance matrix adaptation can be applied to guard against the effects of body accelerations and magnetic disturbances, as discussed in, e.g., [12,23,24]. They are not implemented here.

The state-transition matrix **F***_k_*_−1_, the process noise covariance matrix **Q***_k_*_−1_ and the measurement noise covariance matrix **H***_k_* are dependent on the state. Since the true state is unknown, the state predicted in the prediction stage is used in its place, as it is common practice in EKF formulations. Finally, the unit-quaternion constraint needed for the quaternion to represent a valid rotation is enforced by brute-force normalization after the update stage [10].

The filter initialization is carried out in conditions when the IMU is still and no time-varying magnetic dipoles influence the local magnetic field. The sensor measurements are averaged during an initial rest period of 1 s. The inclination is computed by processing the initial average vector of acceleration measurements. The local magnetic field is estimated by projecting the initial average vector of magnetic measurements using the estimated inclination; the magnetic distortion is initialized to zero. Finally, the initial quaternion and its covariance matrix are computed using the TRIAD method [25]. Computing the average vector of gyro measurements in the rest period allows performing the capture of the gyro-bias, which is then initialized to zero.

### 2.5. Filter Adaptation

The default operating mode of the proposed algorithm is based on the GM-1 model for the magnetic distortion (quiescent EKF). When necessary, extra components are added to the system's state vector, as prescribed by the GM-2 model (higher-order EKF). The higher-order EKF is reverted to the quiescent EKF by another decision. When the magnetic perturbations are small or negligible, using the higher-order EKF increases the state estimation errors. Conversely, using the quiescent EKF may yield maximum estimation accuracy—no information is wasted on estimating components that are zero or small. However, if fast changes of magnetic direction and intensity are experienced, the quiescent EKF may not effectively track the magnetic distortion, which is essential for accurate orientation determination.

To implement the VSD-EKF we apply the technique discussed in [26]. The switching from the quiescent to the higher-order EKF is based on computing a fading memory average of the normalized innovation squared from the quiescent EKF (*a* = 0.9). The switching takes place when the fading memory average of the normalized innovations exceeds a given up-crossing threshold. The fading memory has an effective window length *s*, and the onset of the magnetic perturbation is taken at the beginning of this window. As for the initialization of the higher-order EKF, when a magnetic perturbation is detected at time *k*, the algorithm assumes that the magnetic distortion has a constant rate of change starting at *k* − *s* − 1. Then, the higher-order EKF equations reprocess the IMU measurements sequentially up to time *k*, before sequentially processing incoming measurements at later times. The scheme for reverting to the quiescent EKF is based on using a fading memory average of the normalized innovation squared computed for the extra-state components of the GM-2 model; the fading memory average is then compared to a given down-crossing threshold. When falling below the threshold the quiescent EKF is called on again.

### 2.6. Experimental Validation Tests

The experimental validation tests were carried out using the Xsens MTx orientation sensor, whose raw data were delivered to a host computer via a USB interface at a rate of 100 Hz. The manufacturer expressed the measurements of the magnetic field in arbitrary units (a.u.).

Before starting each test the IMU sensors were calibrated [19,20]. During the static test, whose duration was 3 min, the IMU remained on a table far from electromagnetic devices and ferromagnetic materials. A magnetic disturbance was generated at the time when a cell phone was moved close to the IMU toward the end of the first and second minute of data recording, for approximately 10 seconds. The dynamic test was performed as follows. The IMU was fastened to a plastic plate with size 10 cm × 13 cm using double-side adhesive tape. The plate was raised by the experimenter slightly over the table and then it was freely moved around, thereby sweeping a volume of about 60 cm × 60 cm × 60 cm. The plate orientation was recorded using a six-camera Vicon optical motion analysis system with a sampling rate of *f_s_* = 100 Hz. The Vicon system measured the position of four reflective markers (diameter: 14 mm) arranged at the corners of the plate. The IMU and Vicon data streams were electronically synchronized. The initial orientation of the sensor frame, *i.e.*, the B-frame, relative to the marker frame, *i.e.*, the E-frame, was computed during the initial rest period of the IMU. Occasionally the IMU was moved close to a loudspeaker magnet placed on the table, a first time after 30 seconds of data recording. In the neighborhood of the loudspeaker, the magnetic field was strongly perturbed. The IMU was either taken at rest by the experimenter for few seconds, or it was moved around in the surroundings before being kept away. The visits to the region where the magnetic field was perturbed occurred every minute for approximately 10 seconds.

### 2.7. Performance Assessment and Filter Parameter Tuning

The ground-truth orientation was obtained by processing the marker coordinates provided by the optical motion analysis system as described in [8]. The Euler angles time functions delivered by the EKFs were obtained from the estimated quaternion using standard conversion formulas. We then computed the RMS errors incurred by the EKF in estimating the Euler angles using the ground-truth orientation as reference.

The magnetic field was estimated directly from the magnetic sensor measurements during the static test, since we assumed that the E-frame and the B-frame were aligned; as for the dynamic test, the ground-truth orientation was used to project the magnetic sensor measurements from the B-frame into the E-frame, yielding a good approximation to the magnetic field (ground-truth magnetic field). The strength of the magnetic perturbation was computed by taking the difference vector between the ground-truth magnetic field and the initial field **h**. The overall RMS value was then computed using the root sum-of-square rule applied to single components of the difference vector. Either for static or dynamic tests, the error between the estimated and the ground-truth fields was analyzed in terms of the overall RMS error, computed as described above for the strength of the magnetic perturbation.

The following filter implementations were assessed: the VSD-EKF, the quiescent EKF and the higher-order EKF. Either the quiescent or the higher-order EKF were made adaptive, although we did not develop specific switching rules for adaptation. Our approach was to adapt their state-transition and process noise covariance matrices by changing the value of the parameters valid for the condition Perturbation OFF to those valid for the condition Perturbation ON, or vice versa, using the OFF-ON and ON-OFF switching times computed by the VSD-EKF. For the purpose of a fair comparison between the various filter implementations, the OFF-ON switching times for adapting the quiescent and the higher-order EKFs were taken at the beginning of the fading memory window used by the VSD-EKF to reprocess the IMU measurements. The standard deviation of the random-walk model for gyro-bias compensation was set to *^g^σ* = 0.01 °/s^2^ (filtering option: Y); the gyro-bias compensation was disabled by setting *^g^σ* = 0 °/s^2^. The filter parameter setting reported in Tables 1 and 2 was chosen during the static test the process noise covariance matrix Q was tuned using the value of the gyro measurement noise standard deviation, assessed when the IMU was at rest.

The Kalman-based filters were written in Matlab for off-line data processing using a MacBook Pro computer and the virtualization technology from Parallel Desktop 4.0 for Mac. The VSD-EKF cycle time for a single iteration was about 4 ms, on average.

## 3. Results and Discussion

**Figure 1.**
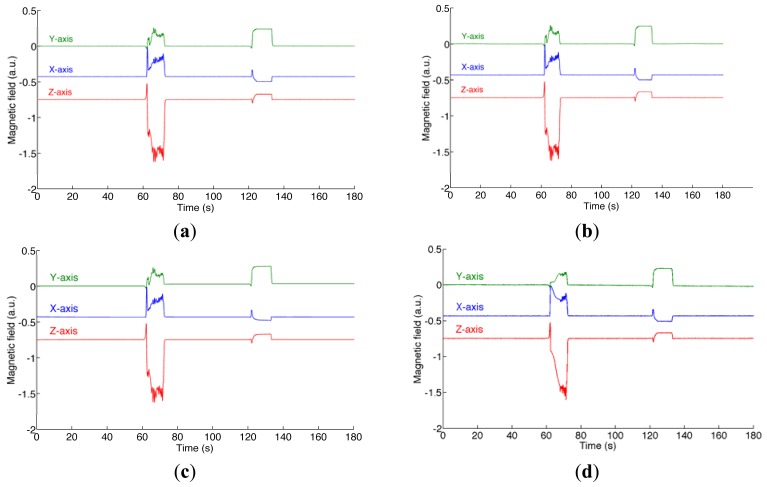
Static test. (**a**) The ground-truth magnetic field; (**b**) The magnetic field estimated by the VSD-EKF; (**c**) the magnetic field estimated by the higher-order EKF; (**d**) the magnetic field estimated by the quiescent EKF.

During the static test the variation of the magnetic field intensity and of the dip angle, namely the angle that the total field vector makes with respect to the horizontal plane, are about 62% and 31%, respectively); the RMS value of the magnetic distortion is about 181 a.u.. Figure 1 shows the ground-truth magnetic field and the magnetic field estimated by the various filters.

**Figure 2.**
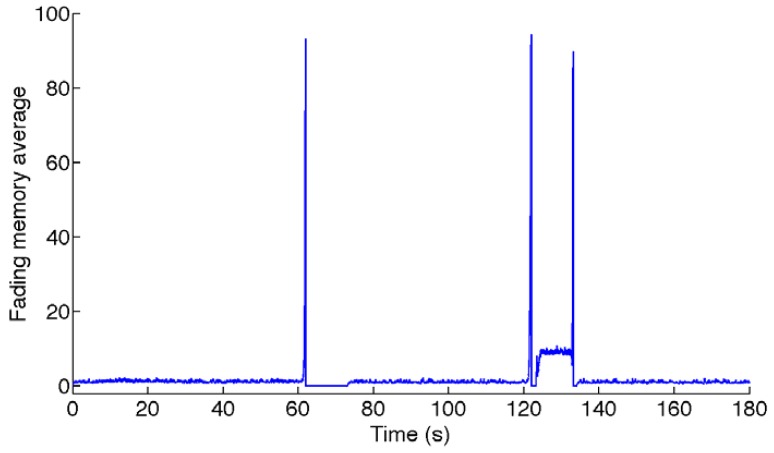
Fading memory average used for switching from the quiescent state model to the higher-order state model in the VSD-EKF (static test).

**Figure 3.**
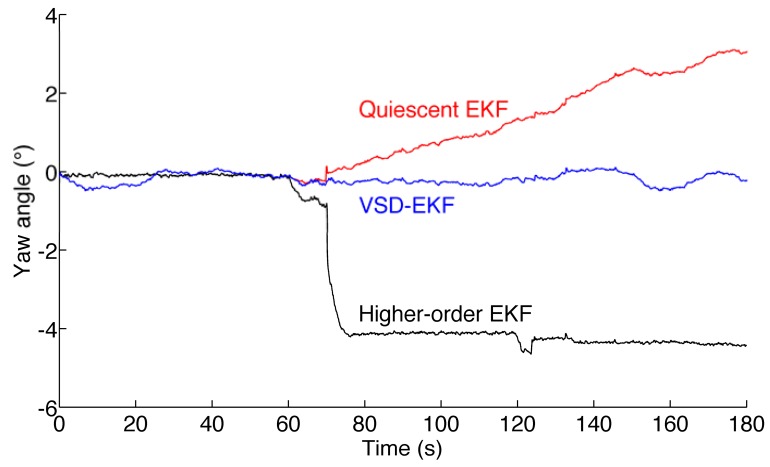
Estimated yaw angle for the three different filters (static test).

The fading memory average that is monitored for switching to the higher-order state model is shown in Figure 2. The sojourns in the higher-order state model are in the intervals *T*_1_ = [61.98; 73.2] s; *T*_2_ = [121.9; 123.4] s; *T*_3_ = [133.1; 134.1] s, where the fading memory average is zero. The RMS errors are reported in Table 3. The estimated yaw angle is plotted in Figure 3.

**Figure 4.**
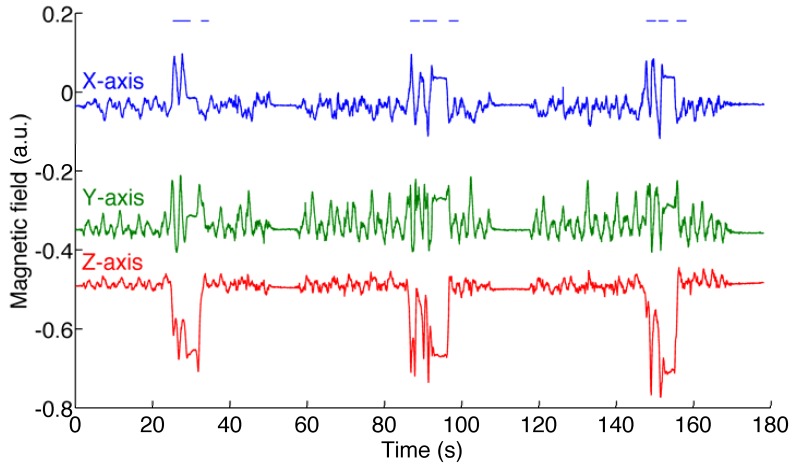
Components of the ground-truth reference magnetic field. The horizontal bars at the top of the plot show the time intervals of sojourns in the higher-order state model (dynamic test).

**Figure 5.**
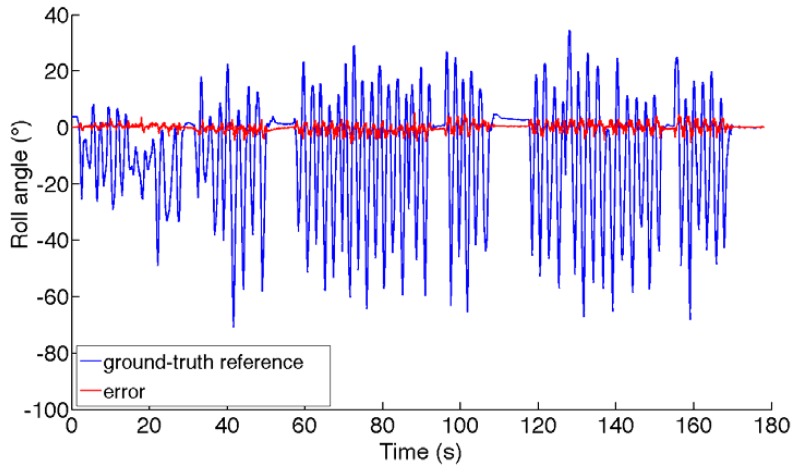

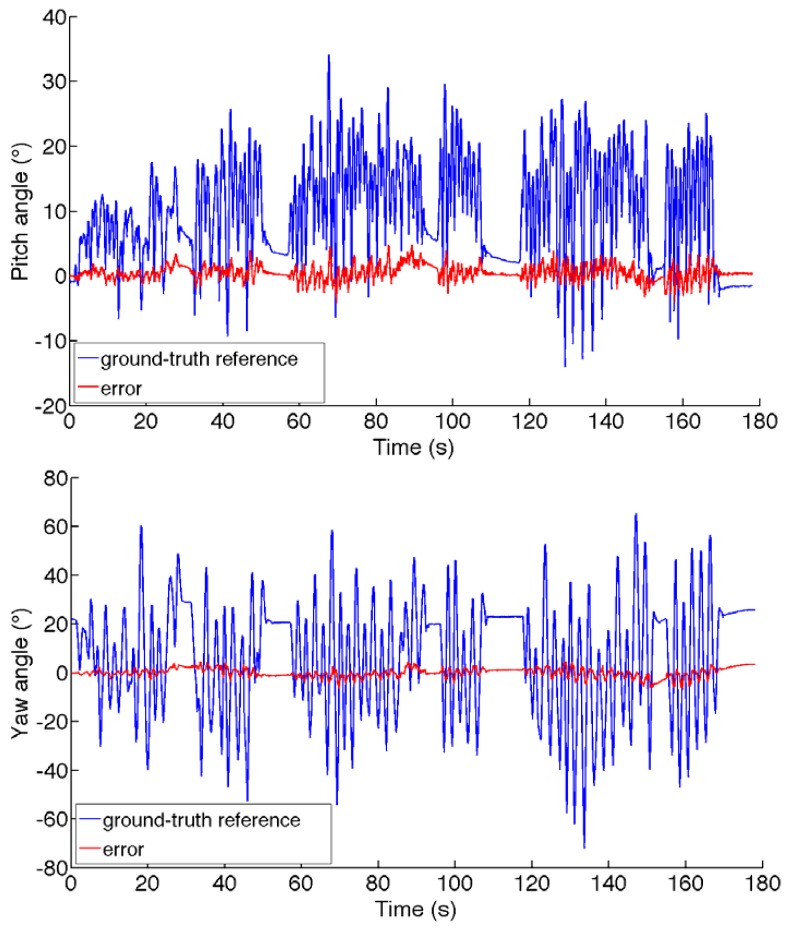
Ground-truth Euler angles and estimation errors incurred by the VSD-EKF (dynamic test).

The dynamic test is carried out in an environment where the RMS value of the magnetic distortion is about 75 a.u. (the variation of the magnetic field intensity and of the dip angle are about 20% and 30%, respectively). The acceleration magnitude is 1 *g* ± 84 m*g*. The ground-truth magnetic field is plotted in Figure 4. The RMS errors are reported in Table 4. Finally, the ground-truth Euler angles and the angle estimation errors are shown in Figure 5.

## 4. Conclusions

The GM-1 model is used in most reported Kalman-based-filters for explaining the magnetic distortion, either in fixed-parameter or adaptive implementations. The filter response to a magnetic perturbation is faster and more accurate when the standard deviation of the process noise is increased and, although to a more limited extent, its correlation time constant is decreased [16]. However, the GM-1 model suffers from severe limitations when abrupt changes of intensity or direction of the perturbed magnetic field have to be dealt with, as the plot of Figure 1 and the results presented in Table 3 show. Since both the disturbance strength and the corresponding variation in the dip angle are of much greater extent during the perturbation at the first minute of data recording as compared with the perturbation at the second minute, the yaw estimate is more affected during the former episode.

Quite typically, the yaw estimates produced by the adapted quiescent EKF may drift even when quiet magnetic conditions are restored after a disturbance occurrence. This is due to its limited capability of accurately tracking the time-changes of the magnetic field when they occur. The inability of the adapted higher-order EKF to produce accurate yaw angle estimates in magnetically perturbed conditions is quite surprising, in the face of the success met by the SVD-EKF, whose model in magnetically perturbed conditions is essentially the same. A possible explanation is that the higher-order EKF is extremely sensitive to the strength of the GM-2 driving noise. In conditions of magnetic quiet, the higher-order EKF tends to diverge when the driving noise strength is too large. With those values of the driving noise strength that are acceptable in conditions of magnetic quiet, the algorithm tends to perform very poorly when the magnetic field is about to change. This does not occur with the SVD-EKF, which relies on the quiescent EKF in conditions of magnetic quiet. As expected, the switching to the higher-order EKF increases the algorithm capability of accurately tracking the time-changes of the magnetic field. In conclusion, we catch the best by combining the two models together as done in the VSD-EKF, which shows good accuracy of the estimated yaw and good compensation of the magnetic disturbances.

The results of the dynamic test show the effectiveness of the proposed VSD-EKF and basically confirm the results of the static test. Not surprisingly perhaps, the RMS errors affecting the roll and pitch angle estimates are worse than in the static test. A possible reason may be due to slight imperfections in the synchronization process between IMU and Vicon data streams and errors incurred by the optical motion analysis system itself in generating the ground-truth reference orientation data used for computation. Another reason is certainly that the acceleration measurements cannot be explained by gravity alone. Although slow in nature, the IMU motion determines additional components in the acceleration measurements that cannot be taken into account when they are used in the measurement update of the EKF. The trick we choose to mitigate this problem is to increase the values of the accelerometer measurement noise standard deviation in the matrix **R**, as compared with the value used during the static test, Table 2. Vector selection schemes are not considered in this paper as a means to guard against the effects of body motions. In our ongoing research on using the VSD-EKF to process the measurements from the inertial/magnetic sensors embedded in a state-of-the-art smartphone, we are planning to investigate how vector selection methods can be exploited in the case of faster body motions than those considered in the present work.

Another interesting observation from our tests is that the gyro-bias compensation generally helps produce more accurate estimates of either orientation or magnetic field. This may be quite surprising, since the gyro output is submitted to a bias capture procedure during the filter initialization and the gyro bias cannot change significantly over the limited time duration of the tests. Our interpretation is that adding a limited amount of pseudo-noise to the gyro-bias components of the state vector has a useful stabilizing effect, which is beneficial in producing more accurate estimates. The injection of limited amounts of process noise is often suggested as a means to stabilize the behavior of Kalman-based filters [27]. However, the stabilizing effect of pseudo-noise injection fails to act in the higher-order EKF. Our explanation is in the reduced observability of the filter design when the time-rate of change of the magnetic field is also included in the system's state.

The results of the tracking tests reported in this paper are quite representative of the behavior of the investigated filters. We may state that the quiescent EKF with gyro bias compensation and the higher-order EKF without gyro bias compensation perform similarly, and both are inferior to the SVD-EKF, sometimes remarkably.

In conclusion, we have presented in this paper an adaptive variable-state-dimension EKF for processing the data from an IMU integrated with a magnetic sensor. The proposed algorithm estimates the quaternion of rotation and attempts to compensate magnetic disturbances and gyro bias. The filter switches from a quiescent EKF, built around a first-order Gauss-Markov model for describing magnetic distortions, to a higher-order EKF, built around the same stochastic model applied to the time-rate of change of the local magnetic field. Experimental validation tests show the effectiveness of the proposed approach in magnetically perturbed environments.

## Figures and Tables

**Table 1.** Parameter tuning.

**Static test**		
**Filtering algorithm**	**Models for magnetic distortion**	
	Perturbation OFF	Perturbation ON
**VSD-EKF**	GM-1	GM-2
Correlation time constant, s Standard deviation	0.1 5 · 10^−2^ a.u./s	0.2 5 · 10^−1^ a.u./s^2^
**Higher-order EKF**	GM-2	GM-2
Correlation time constant, s Standard deviation, a.u./s^2^	0.1 5 · 10^−5^	0.2 5 · 10^−1^
**Quiescent EKF**	GM-1	GM-1
Correlation time constant, s Standard deviation, a.u./s	0.1 5 · 10^−2^	0 5
**Dynamic test**		
**Filtering algorithm**	**Models for magnetic distortion**	
	Perturbation OFF	Perturbation ON
**VSD-EKF**	GM-1	GM-2
Correlation time constant, s Standard deviation	0.1 5 · 10^−2^ a.u./s	0.2 5 · 10^−2^ a.u./s^2^
**Higher-order EKF**	GM-2	GM-2
Correlation time constant, s Standard deviation, a.u./s^2^	0.1 5 · 10^−5^	0.2 5 · 10^−1^
**Quiescent EKF**	GM-1	GM-1
Correlation time constant, s Standard deviation, a.u./s	0.1 5 · 10^−2^	0.4 5

**Table 2.** Parameter tuning.

**Measurement noise standard deviation**	**Static test**	**Dynamic test**

*σ_g_*, °/s	0.4	0.5
*σ_a_*, m*g*	1.0	15.0
*σ_m_*, a.u. (×10^−3^)	1.0	2.0

**Table 3.** Euler angles and magnetic distortion RMSE (static test).

	**Roll, °**	**Pitch, °**	**Yaw, °**	**Field, a.u.**
VSD-EKF-Y	0.09	0.02	0.17	7.89
VSD-EKF-N	0.09	0.02	3.16	25.31
Higher-order EKF-Y	0.07	0.08	3.54	26.10
Higher-order EKF-N	0.07	0.08	2.57	20.81
Quiescent EKF-Y	0.09	0.02	1.60	60.40
Quiescent EKF-N	0.09	0.02	4.87	70.12

**Table 4.** Euler angles and magnetic distortion RMSE (dynamic test).

	**Roll, °**	**Pitch, °**	**Yaw, °**	**Field, a.u.**
VSD-EKF-Y	1.39	1.21	1.90	19.78
VSD-EKF-N	1.81	1.47	6.66	33.02
Higher-order EKF-Y	2.19	1.47	8.20	49.39
Higher-order EKF-N	1.99	2.06	2.18	36.89
Quiescent EKF-Y	1.29	1.10	2.06	20.96
Quiescent EKF-N	1.66	1.27	7.17	37.10
